# Epidemiological and genomic characteristics of global *bla*NDM-carrying *Escherichia coli*

**DOI:** 10.1186/s12941-024-00719-x

**Published:** 2024-06-21

**Authors:** Changyu Xia, Ruyu Yan, Chang Liu, Junbin Zhai, Jie Zheng, Wei Chen, Xiaoli Cao

**Affiliations:** 1 Department of Laboratory Medicine, Peking University First Hospital, Beijing, China; 2 Department of Laboratory Medicine, Nanjing Drum Tower Hospital Clinical College of Nanjing University of Chinese Medicine, Jiangsu, China; 3 Clinical Research Center, the Second Hospital of Nanjing, Affiliated to Nanjing University of Chinese Medicine, Senior technologist Zhongshan Road 321, Nanjing, Jiangsu Province 210003 China

**Keywords:** Carbapenem-resistant *Escherichia coli*, *bla*NDM, Serotype, Virulence factors, Sequence types

## Abstract

**Background:**

*Escherichia. coli* is the most frequent host for New Delhi metallo-β-lactamase (NDM) which hydrolyzes almost all β-lactams except aztreonam. The worldwide spread of *bla*NDM-carrying *E. coli* heavily threatens public health.

**Objective:**

This study aimed to explore the global genomic epidemiology of *bla*NDM- carrying *E. coli* isolates, providing information for preventing the dissemination of such strains.

**Methods:**

Global *E. coli* genomes were downloaded from NCBI database and *bla*NDM was detected using BLASTP. Per software was used to extract meta information on hosts, resources, collection data, and countries of origin from GenBank. The sequence types (STs) and distribution of antimicrobial resistance gene (ARG) were analyzed by CLC Workbench; Plasmid replicons, serotypes and virulence genes (VFs) were analyzed by submitting the genomes to the websites. Statistical analyses were performed to access the relationships among ARGs and plasmid replicons.

**Results:**

Until March 2023, 1,774 out of 33,055 isolates collected during 2003–2022 were found to contain *bla*NDM in total. Among them, 15 *bla*NDM variants were found with *bla*NDM-5 (74.1%) being most frequent, followed by *bla*NDM-1 (16.6%) and *bla*NDM-9 (4.6%). Among the 213 ARGs identified, 27 *bla*CTX-M and 39 *bla*TEM variants were found with *bla*CTX-M-15 (*n* = 438, 24.7%) and *bla*TEM-1B (*n* = 1092, 61.6%) being the most frequent ones, respectively. In addition, 546 (30.8%) plasmids mediated *ampC* genes, 508 (28.6%) exogenously acquired 16 S rRNA methyltransferase encoding genes and 262 (14.8%) *mcr* were also detected. Among the 232 distinct STs, ST167 (17.2%) were the most prevalent. As for plasmids, more than half of isolates contained IncFII, IncFIB and IncX3. The VF *terC*, *gad*, *traT* and *iss* as well as the serotypes O101:H9 (*n* = 231, 13.0%), O8:H9 (*n* = 115, 6.5%) and O9:H30 (*n* = 99, 5.6%) were frequently observed.

**Conclusions:**

The study delves into the intricate relationship between plasmid types, virulence factors, and ARGs, which provides valuable insights for clinical treatment and public health interventions, and serves as a critical resource for guiding future research, surveillance, and implementation of effective strategies to address the challenges posed by *bla*NDM-carrying *E. coli*. The findings underscore the urgent need for sustained global collaboration, surveillance efforts, and antimicrobial stewardship to mitigate the impact of these highly resistant strains on public health.

**Supplementary Information:**

The online version contains supplementary material available at 10.1186/s12941-024-00719-x.

## Introduction

*Escherichia coli*, a rod-shaped, gram-negative bacterium, predominantly resides in the lower intestinal tract of warm-blooded animals, including humans. Known as one of the most frequent opportunistic pathogens, it is a leading cause of urinary, bloodstream, and wound infections in both community and hospital settings. Within the realm of antibiotic resistance, a significant concern is the emergence of New Delhi metallo-β-lactamase (NDM), a member of the β1 metallo-β-lactamase class, capable of hydrolyzing almost all β-lactams except monobactams. It’s initially identified in a Swedish patient in New Delhi, India, in 2008 [1]. At present, the *bla*NDM variants have now spread across more than 60 species in 11 bacterial families, with *E. coli* being the predominant carrier of the *bla*NDM gene [2]. Strains belonging to the ST167, ST410, and ST617 lineages are the most prevalent clones [3, 4]. Geographically, the Indian subcontinent, the Middle East, and the Balkans are the most epidemic regions [5]. The global dissemination of *bla*NDM-carrying strains poses a considerable challenge for clinical management and public health, due to the heightened mortality rates associated with infections caused by these strains. Besides, various antimicrobial resistance genes (ARGs), such as non-NDM carbapenem hydrolyzing β-lactamases (CHβLs), extended-spectrum β-lactamases (ESBLs), plasmid-mediated quinolone resistance genes (PMQRs), and exogenously acquired 16 S rRNA methyltransferase (16 S-RMTase) genes, are often co-harbored with *bla*NDM in *E. coli*, leading to multi-drug resistance or pan-drug resistance [6, 7], thereby limiting antimicrobial treatment options for *E. coli* infection in clinical settings.

The escalating prevalence of *bla*NDM worldwide can be attributed to global travel and extensive antibiotic use, recognized as key population risk factors linked to the dispersal of *bla*NDM. Notably, the spread of *bla*NDM genes is primarily facilitated by mobile genetic elements (MGEs), with plasmids being the most common carriers. These *bla*NDM-carrying plasmids typically fall under limited replicon types, such as IncX3, IncFII, or IncC [8]. Despite numerous studies on the epidemiological characteristics of Carbapenem-resistant *E. coli* (CREC), comprehensive data on the virulence factors (VFs), serotypes, and sequence types (STs) of *bla*NDM-carrying *E. coli* remain limited.

This study aims to characterize the global epidemiological features of *bla*NDM-carrying *E. coli* by leveraging genomic data from GenBank. The investigation includes an analysis of the distribution of other ARGs, plasmid replicons, VFs, serotypes, and STs to provide a thorough genomic characterization. Additionally, the study explores the consistency in the distribution of plasmid replicons and VFs, as well as VFs and ARGs, shedding light on potential associations between these key elements.

## Materials and methods

### Download of *E. coli* genome data

Sequence files of all 33,055 *E. coli* genomes (updated to 2023.03.09) were downloaded in batch from NCBI (https://www.ncbi.nlm.nih.gov/genome/browse/#!/overview/) using the aspera high-speed download tool. For all annotated genomes, the protein coding gene sequence of each genome is obtained in batch from GenBank file by using self-made Perl script. All genomes were qualified with completeness > 90%, contamination < 5%, and contig quantity ≤ 500 and N50 ≥ 40,000.

### *blaNDM* identification

All *bla*NDM sequences were obtained from the NCBI Biological Resistance Reference Gene Database (https://www.ncbi.nlm.nih.gov/pathogens/refgene/#gene_family:(blaNDM). BLASTP was performed, with thresholds being set as expected value = 1e-5, coverage ≥ 60%, identity ≥ 90%, and match length = subject gene length. Finally, the results were processed by Perl program to obtain the detailed distribution of *bla*NDM gene in *E. coli* genomes.

### Extraction of meta-information on the *blaNDM*-carrying *E. coli*

Meta information on hosts, resources, collection data, and countries of origin was extracted from GenBank using Per software. This information was integrated with the ARGs, STs, VFs and serotypes into the same excel for further analysis.

### Investigation into the prevalence of antimicrobial resistance genes

The distribution of other ARGs was investigated using CLC workbench version 21.0.1. The fasta file was input into the files in CLC Workbench using standard import, and after the consensus sequence was extracted, the prevalence of ARGs was analyzed using the ResFinder database for comparison. The results were exported as scv files, and further sorted for analysis.

### Sequence types

STs of *bla*NDM-carrying *E. coli* were identified using CLC workbench 21.0.1. After consensus sequences were extracted, STs were analyzed using multi-locus sequence typing (MLST) with *E. coli* (Oxford) as the reference database.

### Distributions of plasmid replicons, VFs, and serotypes

Genomes were submitted to the Center for Genomic Epidemiology (http://www.Genomicepidemiology.org/). Plasmid Finder 2.1 was used to identify plasmid replicons, and VFs were analyzed by Virulence Finder 2.0. Moreover, serotypes were identified by Serotype Finder 2.0.

### Statistical analyses

Correlation analyses were performed using SPSS 22.0. The distribution consistency of VFs and ARGs was tested by McNimar analysis, and a p-value of > 0.05 was taken as the consistency between them.

## Results

### The prevalent characteristics of *blaNDM*-carrying *E. coli*

**Fig. 1:**
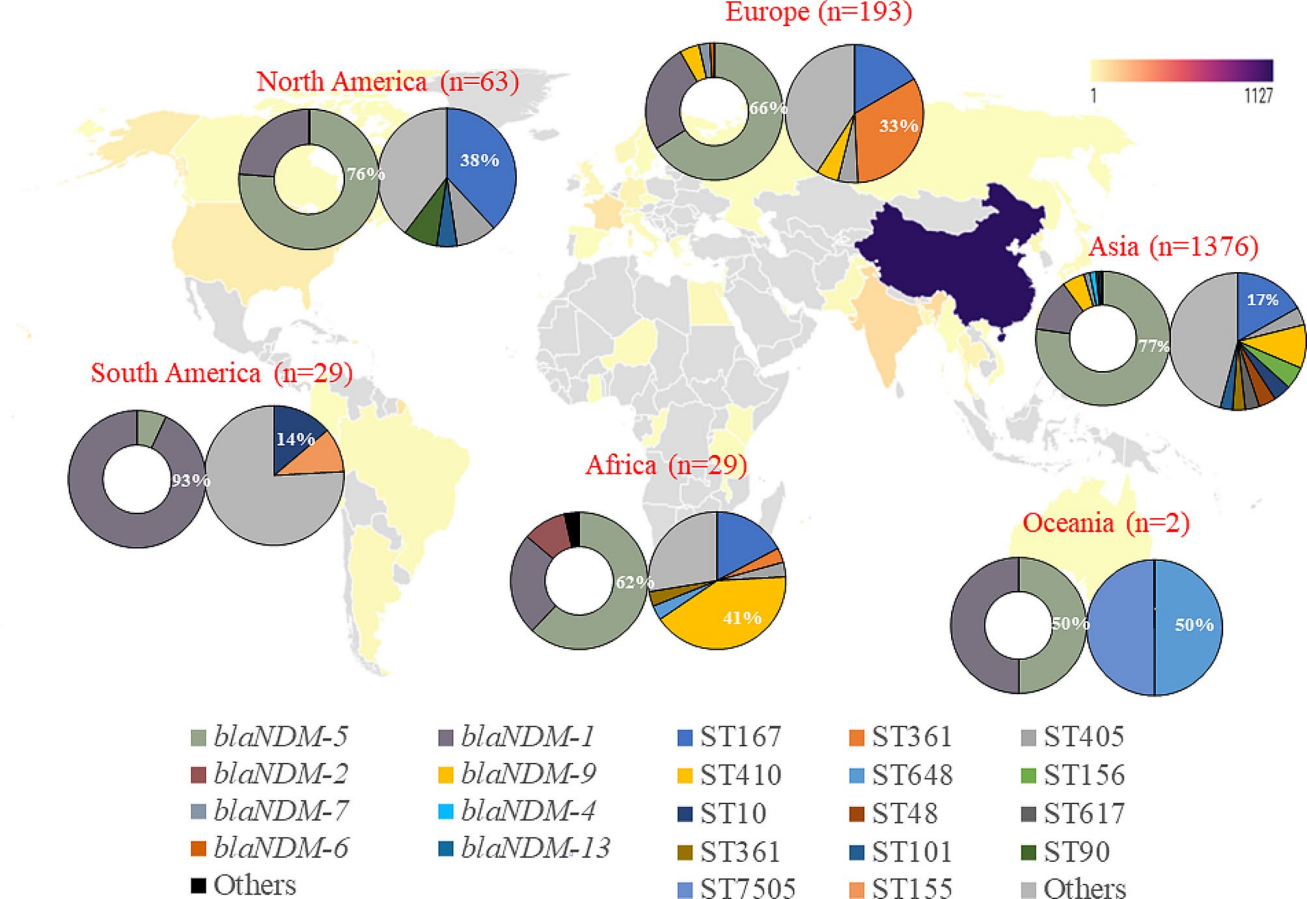
Geographical distribution of *blaNDM* and STs of *bla*NDM-carrying *Escherichia coli* worldwide. Hollow and solid pie charts of each continent represent *bla*NDM and STs, respectively

Totally, 33,055 *E. coli* isolates were downloaded from NCBI database and the time span was from 2003 to 2022. Of which, 1,774 were identified to be positive for the *bla*NDM gene (Additional file 1). These isolates were obtained from 43 countries across 6 continents (Fig. 1), which were as follows: Asia (*n* = 1376, 77.6%), Europe (*n* = 196, 11.0%), North America (*n* = 63, 3.6%), Africa (*n* = 29, 1.6%), South America (*n* = 29, 1.6%), and Oceania (*n* = 2, 0.1%). Of concern, China (*n* = 1127, 63.5%), India (*n* = 115, 6.5%), and France (*n* = 83, 4.7%) were the primary contributors, submitting the highest number of genomes, following closely were the USA (*n* = 56), Germany (*n* = 33), Thailand (*n* = 30), United Kingdom (*n* = 21), Bangladesh (*n* = 20), South Korea (*n* = 19), Lebanon (*n* = 16), and Pakistan (*n* = 11). The origin of the remaining 79 isolates was unspecified.

**Fig. 2:**
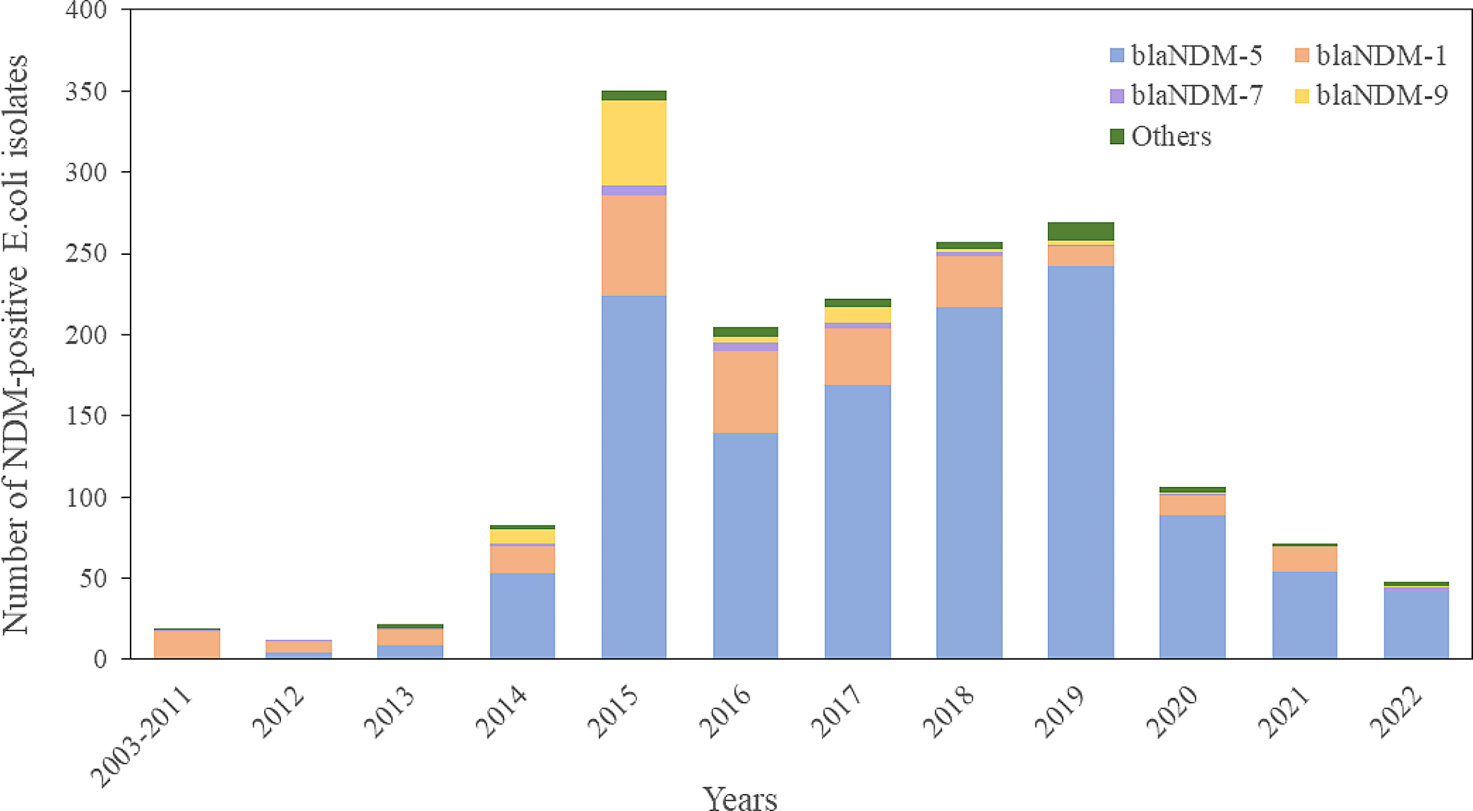
Number of global *bla*NDM-carrying *Escherichia coli* isolates submitted per year

Of 1,774 *bla*NDM genes, 15 distinct *bla*NDM variants were identified. While the number of *bla*NDM-carrying *E. coli* showed a gradual increase each year, it surged significantly in 2015 (*n* = 350). This elevated level was sustained from 2016 to 2019, followed by a decline from 2020 to 2022 (Fig. 2).

Regarding the sources of the *bla*NDM-carrying *E. coli* isolates (Table 1), it was observed that Homo sapiens accounted for the majority, constituting 59.6% (*n* = 1,057) of the total. These isolates were predominantly sourced from urine (*n* = 232), blood (*n* = 162), rectal/anal swabs (*n* = 149), sputum (*n* = 81), and fecal samples (*n* = 86). Animals comprised 27.4% (*n* = 486) of the isolates, with chickens (*n* = 182), pigs (*n* = 59), and flies (*n* = 46) being the most prevalent species. The primary sources for animal isolates included fecal samples, cloaca swabs, and various organs (intestine/liver/other). Notably, *bla*NDM-carrying strains were also detected in the environment, accounting for 13.0% (*n* = 231) of the isolates. These environmental sources included water, hospitals, and various food items.

**Table 1:** The hosts and sample types of global *bla*NDM-carrying *E. coli* isolates

Hosts (*n*)		Sample types (*n*)
Homo sapiens (1057)		Urine (232), blood (162), rectal/anal swab (149), fecal sample (86), sputum (81), bile (13), catheter tip (5), ear swab (1), vaginal swab (1), wound/other body fluids/pus (133), NA (194)
Animals (486)	Chicken (182)	Fecal sample (63), cloaca swab (83), intestine/liver/other organs (30), NA (8)
	Pig (59)	Fecal sample (50), NA (9)
	Fly (46)	Fecal sample (46)
	Swine (42)	Fecal sample (38), NA (4)
	poultry (37)	Fecal sample (33), Droppings (2), NA (2)
	Waterfowl (27)	NA (27)
	Others (93)	Fecal sample (35), Ear swab (2), liver (2), NA (54)
Environments (231)		Water (28), hospital (20), Milk (2), environment (13), leaf rape (1), medical sewage (1), NA (166)

NA, not applicable

### Wide distribution of various resistance genes among *blaNDM*-carrying *E. coli*

Among the 1,774 *bla*NDM-carrying isolates, 15 distinct variants were identified, with *bla*NDM-5 being the most prevalent (*n* = 1,315, 74.1%), followed by *bla*NDM-1 (*n* = 295, 16.6%) and *bla*NDM-9 (*n* = 82, 4.6%). Other variants included *bla*NDM-7, *bla*NDM-4, *bla*NDM-6, *bla*NDM-13, *bla*NDM-3, *bla*NDM-15, *bla*NDM-19, *bla*NDM-16, *bla*NDM-20, *bla*NDM-21, *bla*NDM-22, and *bla*NDM-24, each with lower frequencies. Geographically, *bla*NDM-5 dominated in Asia, Europe, Africa, and North America, constituting 62.0-77.0% of cases (Fig. 1). Notably, South America exhibited a distinct pattern, with *bla*NDM-1 being the most common variant, representing 93.0% of cases.

**Fig. 3:**
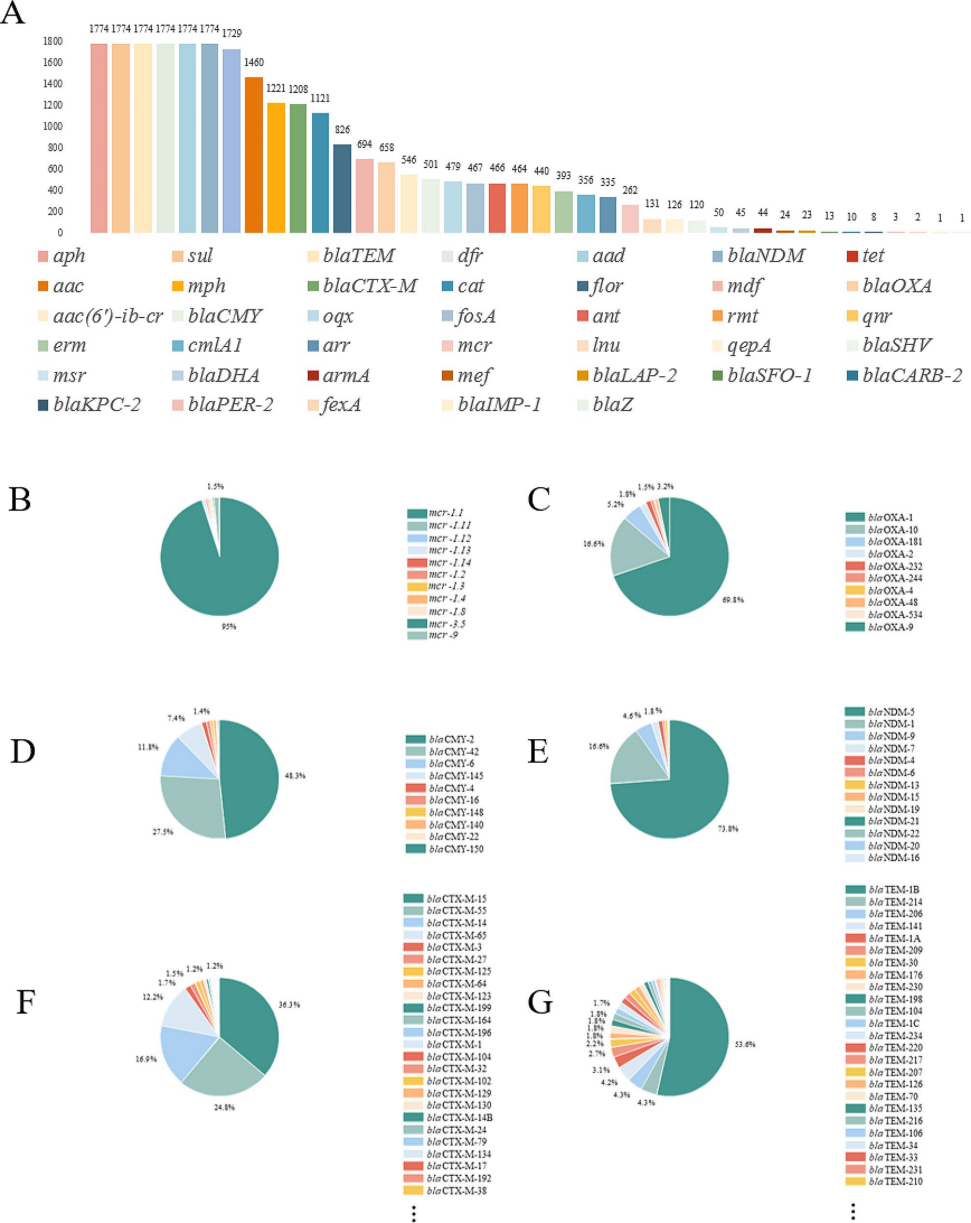
The antimicrobial resistance genes identified among 1774 *bla*NDM-carrying *Escherichia coli*. 3 **A**. all the antimicrobial resistance genes; 3**B**. *mcr* variants; 3 **C**, *bla*OXA variants; 3**D**. *bla*CMY variants; 3**E**. *bla*NDM variants; 3 **F**. *bla*CTX-M variants; 3**G**. *bla*TEM variants

A comprehensive analysis of ARGs in *bla*NDM-carrying strains revealed 213 different types. Among them, CHßLs encoding genes including 8 *bla*KPC-2, 1 *bla*IMP-1, 34 *bla*OXA-181, 10 *bla*OXA-232, 5 *bla*OXA-244 and 5 *bla*OXA-48 were identified. Moreover, 27 *bla*CTX-M and 39 *bla*TEM variants were detected with *bla*CTX-M-15 (n = 438, 24.7%), *bla*CTX-M-55 (n = 300, 16.9%), *bla*CTX-M-14 (n = 204, 11.5%), *bla*CTX-M-65 (n = 147, 8.3%) and *bla*TEM-1B (n = 1092, 61.6%) being the most frequent ones, respectively. In addition, 546 (30.8%) plasmids mediated *ampC* genes, including 501 *bla*CMY and 45 *bla*DHA as well as 508 (28.6%) 16S-RMTase encoding genes, including 409 *rmtB*, 55 *rmtC* and 44 *armA* were found. Of significant concern, 262 (14.8%) co-existing *mcr* genes were also detected, with *mcr*1.1 being the most prevalent genotype (n = 249, 14.0%). Furthermore, 467 (26.3%) fosfomycin resistance genes including 459 *fosA3* and 8 *fosA4* as well as 546 (30.8%) *aac(6’)-ib-cr* conferring resistance to amikacin and fluoroquinolones in addition to 805 (45.4%) PMQRs including 239 *oqxAB*, 126 *qepA*, and 440 *qnr* were identified. Other main ARGs detected were shown in Fig. 3.

### Multiple distinct sequence types were identified with several high-risk clones being prevalent

A total of 232 distinct STs were identified among the *bla*NDM-carrying *E. coli* isolates. The most prevalent was ST167 (*n* = 306, 17.2%), followed by ST410 (*n* = 174, 9.8%), ST361 (*n* = 108, 6.1%), ST405 (*n* = 85, 4.8%), ST156 (*n* = 74, 4.2%), ST10 (*n* = 73, 4.1%), ST48 (*n* = 59, 3.3%), ST617 (*n* = 53, 3.0%), ST101 (*n* = 50, 2.8%), ST648 (*n* = 44, 2.5%), and ST746 (*n* = 38, 2.1%). Several other STs were also identified, with each less than 30. Geographically, the distribution of STs varied. ST167 was predominant in both Asia and North America, ST361 was endemic in Europe, and ST410 emerged as the predominant clone in Africa, with ST10 dominating in South America (Fig. 1).

### Virulence factors

A total of 170 distinct VFs were identified among the *bla*NDM-carrying *E. coli* isolates. The most prevalent VF was *terC* (*n* = 1,766, 99.5%). Notably, more than half of the isolates carried four specific VFs: *gad* (83.4%, *n* = 1,480), *traT* (67.9%, *n* = 1,205), and *iss* (51.3%, *n* = 910). Several other VFs were also frequently detected, including *sitA* (*n* = 756, 42.6%), *hra* (*n* = 689, 38.8%), *lpfA* (*n* = 631, 35.6%), *fyuA* (*n* = 561, 31.6%), and *irp2* (*n* = 560, 31.6%).

Of significant concern is the differential distribution of the predominantly prevalent VFs among the endemic clones. Notably, *lpfA* was primarily distributed among ST410 and ST156, while *iss* was predominantly concentrated in ST167 and ST156. The dominance of *fyuA* and *irp2* was observed among ST405, with ST361 and ST405 being the most frequent carriers for *sitA*.

### Multiple Plasmid replicons were found among *blaNDM*-carrying *E. coli* which may facilitate the spread of antimicrobial resistance genes

Various plasmid replicons were identified, with IncFII (*n* = 1,163, 65.6%) emerging as the most prevalent. Following closely were IncFIB (*n* = 1,157, 65.2%), IncX3 (*n* = 888, 50.1%), IncFIA (*n* = 756, 42.6%), COL (*n* = 440, 24.8%), IncY (*n* = 338, 19.1%), IncI1-I (*n* = 321, 18.1%), P0111 (*n* = 306, 17.2%), IncHI2 (*n* = 287, 16.2%), IncQ1 (*n* = 194, 10.9%), IncI (*n* = 190, 10.7%), IncFIC (*n* = 179, 10.1%), IncC (*n* = 174, 9.8%), IncR (*n* = 114, 6.4%), IncX1 (*n* = 128, 7.2%), IncN (*n* = 77, 4.3%), and several other rare plasmid replicons.

Analysis revealed that 44.7–66.8% of *bla*NDM-5-carrying strains carried IncFII, IncFIB, IncX3, and IncFIA plasmids (Table 2). For *bla*NDM-1-carrying strains, over half carried IncFII and IncFIB plasmids, with IncFIA and Col being relatively highly prevalent. IncFIB, IncFII, and Col were frequently carried by *bla*NDM-9 positive strains. Notably, IncFIA was particularly prevalent, reaching 80.9%, in *bla*NDM-7-carrying strains, followed by IncFIB, IncFII, and IncX3.

**Table 2:** Plasmids distribution among different *bla*NDM-carrying strains

	IncFII (*n* = 1163)	IncFIB (*n* = 1157)	IncX3 (*n* = 888)	IncFIA (*n* = 756)	Col (*n* = 440)	IncY (*n* = 338)	IncI1-I (*n* = 321)	P0111 (*n* = 306)	IncHI2 (*n* = 287)
***bla*** **NDM-5** (*n* = 1315)	878 (66.8%)	860 (65.4%)	752 (57.2%)	588 (44.7%)	312 (18.2%)	254 (19.3%)	251 (19.1%)	232 (17.6%)	211 (16.0%)
***bla*** **NDM-1** (*n* = 295)	191 (64.7%)	176 (59.7%)	67 (22.7%)	111 (37.6%)	102 (34.6%)	43 (14.6%)	53 (18.0%)	55 (18.6%)	49 (16.6%)
***bla*** **NDM-9** (*n* = 82)	47 (57.3%)	62 (75.6%)	2 (2.4%)	12 (14.6%)	38 (46.3%)	19 (23.2%)	23 (28.0%)	20 (24.4%)	16 (19.5%)
***bla*** **NDM-7** (*n* = 32)	21 (65.6%)	23 (71.9%)	21 (65.6%)	17 (80.9%)	6 (18.8%)	6 (18.8%)	2 (6.2%)	3 (9.4%)	3 (9.4%)

Analyzing the consistency between prevalent plasmids and resistant/virulent genes (Table 3) revealed correlations between the prevalence of *mph(A)* and *tra(T)* and the plasmid replicons IncFII and IncFIB (*p* > 0.05). The incidences of *aph(6)-Id*, *aadA2*, *aph(3’’)-Ib*, *dfrA12*, and *iss* were all correlated with the presence of plasmid IncX3. Additionally, the distribution of *bla*OXA-1, *iucC*, and *capU* was consistent with the presence of plasmid Col.

**Table 3:** Consistency analysis between plasmids and resistant/virulent genes

Genes	IncFII (*n* = 1163)	IncFIB (*n* = 1157)	IncX3 (*n* = 888)	IncFIA (*n* = 756)	Col (*n* = 440)	IncY (*n* = 338)	IncI1-I (*n* = 321)	P0111 (*n* = 306)	IncHI2 (*n* = 287)
*sul1* (*n* = 1234)	0.006	0.000	0.000	0.000	0.000	0.000	0.010	0.000	0.000
*mph(A)* (*n* = 1173)	0.747*	0.588*	0.000	0.000	0.000	0.000	0.000	0.000	0.000
*bla*TEM-1b (*n* = 1092)	0.000	0.000	0.000	0.003	0.000	0.000	0.000	0.000	0.000
*tet(A)* (*n* = 1084)	0.008	0.012	0.000	0.000	0.000	0.000	0.000	0.000	0.000
*aph(6)-Id* (*n* = 913)	0.000	0.000	0.428*	0.000	0.000	0.000	0.000	0.000	0.000
*aadA2* (*n* = 906)	0.000	0.000	0.586*	0.000	0.000	0.000	0.000	0.000	0.000
*aph(3’’)-Ib* (*n* = 898)	0.000	0.000	0.767*	0.000	0.000	0.000	0.000	0.000	0.000
*dfrA12* (*n* = 826)	0.000	0.000	0.053*	0.016	0.000	0.000	0.000	0.000	0.000
*floR* (*n* = 826)	0.000	0.000	0.032	0.028	0.000	0.000	0.000	0.000	0.000
*bla*OXA-1 (*n* = 459)	0.000	0.000	0.000	0.000	0.475*	0.000	0.000	0.000	0.000
*terC* (*n* = 1766)	0.000	0.000	0.000	0.000	0.000	0.000	0.000	0.000	0.000
*Gad* (*n* = 1480)	0.000	0.000	0.000	0.000	0.000	0.000	0.000	0.000	0.000
*traT* (*n* = 1205)	0.093*	0.086*	0.000	0.000	0.000	0.000	0.000	0.000	0.000
*iss* (*n* = 910)	0.000	0.000	0.464*	0.000	0.000	0.000	0.000	0.000	0.000
*sitA* (*n* = 756)	0.000	0.000	0.000	1.000	0.000	0.000	0.000	0.000	0.000
*hra* (*n* = 689)	0.000	0.000	0.000	0.020	0.000	0.000	0.000	0.000	0.000
*lpfA* (*n* = 631)	0.000	0.000	0.000	0.000	0.000	0.000	0.000	0.000	0.000
*fyuA* (*n* = 561)	0.000	0.000	0.000	0.000	0.000	0.000	0.000	0.000	0.000
*irp2* (*n* = 560)	0.000	0.000	0.000	0.000	0.000	0.000	0.000	0.000	0.000
*ompT* (*n* = 530)	0.000	0.000	0.000	0.000	0.001	0.000	0.000	0.000	0.000
*iucC* (*n* = 456)	0.000	0.000	0.000	0.000	0.565*	0.000	0.000	0.000	0.000
*capU* (*n* = 485)	0.000	0.000	0.000	0.000	0.105*	0.000	0.000	0.000	0.000

*p > 0.05 was considered as the consistency between resistant/virulent genes and plasmid replicons

**Fig. 4:**
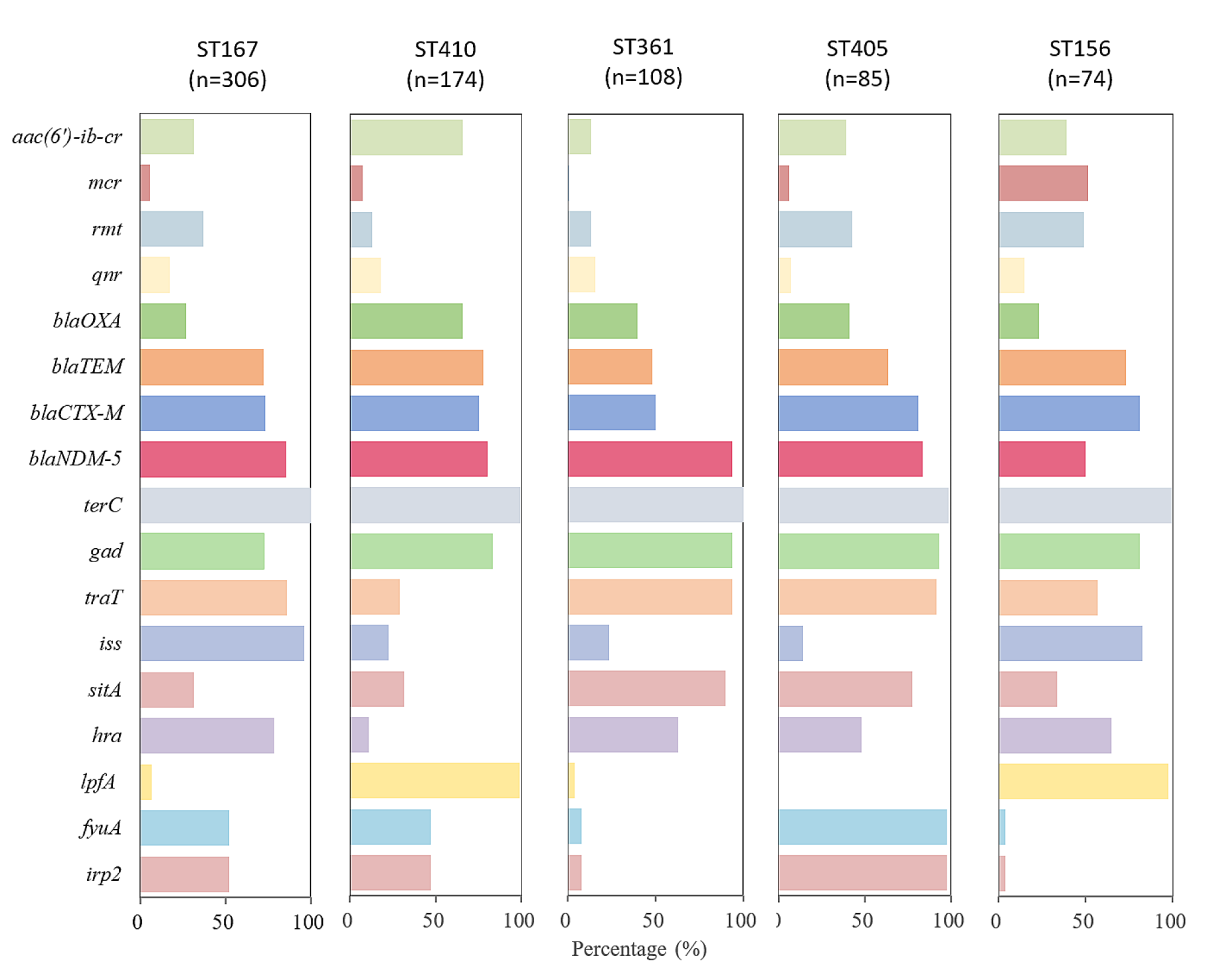
The prevalence of prevalent antimicrobial resistance genes and virulent factors among epidemic clones. Antimicrobial resistance genes were *aac(6’)-ib-cr*, *mcr*, *rmt*, *qnr*, *bla*OXA, *bla*TEM, *bla*CTX-M, *bla*NDM-5; Virulence factors were *terC*, *gad*, *traT*, *iss*, *sitA*, *hra*, *lpfA*, *fyuA*, *irp2*

The prevalence of ARGs and VFs among epidemic clones ST167, ST410, ST361, ST405 and ST156 were demonstrated in the Fig. 4. More than 42.5% of them carried *bla*TEM, *bla*CTX-M, *bla*NDM-5, *traT*, *gad* and *terC*, constituting a basic resistant and virulent profile. In detail, ST167 clones exhibited high incidences of *iss* and *hra*; S156 showed a high carriage of *mcr*, *rmt*, *lpdA, iss,* and *hra*. Notably, ST410 clones demonstrated nearly 100% carriage of *lpfA* and high incidences of *aac(6’)-ib-cr* and *bla*OXA, while ST405 showed a high prevalence of *fyuA*, *irp2*, *sitA*, *traT*, *gad* and *rmt*. This highlights the distinct prevalence patterns of ARGs among different STs, emphasizing the diversity in resistance and virulence profiles associated with specific *E. coli* lineages.

### A large number of serotypes detected among *blaNDM*-carrying *E. coli* with O101:H9 and O8:H9 being the predominate 

A total of 91 distinct O types were identified, with O101 (*n* = 391, 22.0%), O8 (*n* = 179, 10.1%), O9 (*n* = 154, 8.7%), and O102 (*n* = 92, 5.2%) emerging as the dominant ones. Among the 43 H types, H9 (*n* = 470, 26.5%) was the most prevalent, followed by H30 (*n* = 140, 7.9%), H6 (*n* = 130, 7.3%), H10 (*n* = 121, 6.8%), and H5 (*n* = 114, 6.4%). Over 100 distinct serotypes were identified, with O101:H9 (*n* = 231, 13.0%), O8:H9 (*n* = 115, 6.5%), O9:H30 (*n* = 99, 5.6%), O102:H6 (*n* = 86, 4.8%), and O101:H10 (*n* = 77, 4.3%) being the most frequent.

## Discussion

The increasing prevalence of *bla*NDM-carrying *E. coli* presents significant challenges to clinical treatment and global public health, prompting a critical need to bolster infection control measures in hospitals. The epidemiological and genomic characterization of 1,774 *bla*NDM-carrying *E. coli* isolates from 43 countries (2003–2022) provides valuable insights for guiding clinical treatment strategies and implementing preventative measures.

Our study showed that prevalence rates of *bla*NDM-carrying *E. coli* varied globally, with Asia reporting the highest, followed by Europe (11.0%), America (5.2%), Africa (1.6%), and Oceania (0.01%). These rates closely align with a meta-analysis of 110 studies from 2008 to 2018 [9], confirming the high prevalence of such strain in Asia. In addition, we identified a notable surge of *bla*NDM in *E. coli* isolates during 2015–2019, with *bla*NDM-5 emerging as the most frequent variant. Consistent with this, an upward trend in *bla*NDM-carrying *Enterobacterale* has been observed in France since 2012, in Switzerland since 2013, and in Germany from 2013 to 2019 [10–13]. Particularly concerning is the rapid global spread of *bla*NDM-5 producing *E. coli*, contributing to an epidemic situation in India, China, and sub-Saharan Africa [5, 14]. Furthermore, the global prevalence of *bla*NDM experienced a decline in 2020 and 2021, likely attributed to some sequence data not being released at the time of our data retrieval in 2023. Moreover, our study showed that animals and environments constituted 40% of the studied resources, which may relate with the use of carbapenem antibiotics in poultry breeding. Notably, as observed in other studies, there was a substantial prevalence of *bla*NDM-5 in poultry and farm environments, which may be spread from healthcare settings [15].

Of great note, our findings align with previous research, affirming that all *bla*NDM-positive *E. coli* isolates in our study co-carried additional ARGs. The most prevalent among these were *sul1* and *sul2*, encoding an alternative dihydropteroate synthase, emphasizing the need for cautious prescription of sulfonamides targeting CREC. Furthermore, we observed the coexistence of *bla*ESBL, particularly *bla*TEM-1b and *bla*CTX-M-15, with the *bla*NDM-5 gene, consistent with prior reports [12, 16]. This underscores the complexity of resistance profiles and emphasizes the importance of comprehensive antimicrobial stewardship. It is noteworthy that in our study, 61.1% of *bla*NDM-carrying *E. coli* concurrently carried the *tet(A)* gene, known to result in reduced susceptibility to tigecycline, signaling a need for cautious use of tigecycline in treating such strains [17, 18]. Additionally, a significant 14.0% of *bla*NDM-carrying *E. coli* harbored the *mcr*1.1 gene, a horizontally transmitted colistin resistance gene [19]. This finding is of great concern as it poses a challenge to the effectiveness of colistin in treating infections caused by these strains. Further complicating matters, our study identified nine derivatives of *mcr* (*mcr-2* to *mcr-10*) [20], suggesting a potential reduction in polymyxin sensitivity among *bla*NDM-positive strains due to the widespread presence of *mcr*. Furthermore, 46.6% of *bla*NDM-positive strains simultaneously carried the Trimethoprim-resistance gene *dfrA12* and Florfenicol resistance genes *floR*, indicating a multi-drug resistance profile. Notably, in strains carrying both *mcr-1* and *bla*NDM-5, our study revealed IncFIB as the main plasmid carrying both genes, deviating from previous observations [21, 22], possibly due to the extensive inclusion of strains in our analysis. This emphasizes the intricate interplay of resistance mechanisms and highlights the necessity for vigilant antimicrobial management strategies.

ST analysis revealed a diverse array of STs among *bla*NDM-carrying *E. coli*. Notably, ST167 dominated in China and India, while ST361 was predominant in Europe, diverging from previous reports which indicated that ST101 and ST131 are the most prevalent clones in Asia, ST101 and ST405 are the dominant STs in Europe [9]. This discrepancy may arise from the focus on *bla*NDM-carrying *E. coli* in our study, reflecting distinct strain compositions. Consistent with other studies, ST167 and ST410 emerged as international epidemic clones linked with *bla*NDM-5 [23, 24]. Contrary to these findings, no specific ST linkage was observed for *bla*NDM-1 [5], although some strains were assigned to ST410, ST156, ST167, ST10, and ST101. Remarkably, ST13, recognized as the pandemic clone associated with global dissemination of the *bla*CTX-M-15 [25, 26], was identified in only 17 *bla*NDM-carrying *E. coli* isolates, underscoring a divergence in the dissemination patterns of different ARGs.

Among various VFs, over half of the strains carried *terC*, *gad*, *traT*, and *iss*. *terC*, encoding a tellurium iron resistance protein [27]. The *gad* gene, facilitating survival in acidic environments, and the *traT* gene, which encodes protections, along with *iss*, *sitA*, and *hra* genes being associated with urinary tract infection, were also prevalent [28, 29]. Importantly, the distribution of predominant VFs varied significantly among the prevailing clones, highlighting the intricate relationship between genetic backgrounds and virulence traits [30].

IncFII and IncFIB emerged as the predominant plasmids in all *bla*NDM-carrying isolates, showcasing a correlation with the *mph(A)* and *tra(T)* genes. Following closely were IncX3 and IncFIA. Notably, it’s reported that IncX3-type plasmids are recognized as key contributors to horizontal transmission of *bla*NDM in CREC [31] and IncFIB is prevalent especially in *bla*NDM-1-carrying *E. coli* in Greece [32]. Serotype O101, associated with both animal and human diseases, is commonly detected among pathogenic *E. coli* [33]. In our study, O101:H9 emerged as the most prevalent, even though reports of this specific serotype are limited. Interestingly, O101:H9 has been linked to possible international dissemination via migratory birds [34], emphasizing the importance of employing techniques such as multiplex PCR or whole-genome sequencing that go beyond focusing solely on serotype O157:H7 in clinical laboratories. These diverse molecular findings underscore the necessity for ongoing surveillance of *bla*NDM-carrying *E. coli* strains.

To our best knowledge, this study represents the largest comprehensive report on the prevalence and genetic characterization of *bla*NDM-carrying *E. coli* worldwide, utilizing whole-genome data. However, it is crucial to acknowledge certain limitations. Firstly, the inclusion criteria were limited to data available on the NCBI database, potentially introducing selection bias. Secondly, the study’s ability to fully capture the global prevalence of *bla*NDM-carrying *E. coli* is constrained by the insufficient availability of genome data from many countries. Thirdly, the absence of detailed epidemiological data limits the exploration of factors such as prior exposure to healthcare settings and environmental influences, which may play a significant role in the global transmission of *bla*NDM-carrying *E. coli*. Despite these limitations, this study offers valuable insights into the epidemiology and genomic characteristics of *bla*NDM-carrying *E. coli* on a global scale.

## Conclusions

This study offers a comprehensive understanding of the prevalence, genetic diversity, and associated factors of *bla*NDM-carrying *E. coli*, submitted by 43 countries over the period 2003–2022. *bla*NDM-5 is the predominant variant, identified in diverse ST and O: H serotypes, highlighting the complexity and adaptability of these multidrug-resistant strains. The study delves into the intricate relationship between plasmid types, virulence factors, and ARGs, providing valuable insights for clinical treatment and public health interventions. As the global threat of antimicrobial resistance continues to escalate, this study serves as a critical resource for guiding future research, surveillance, and implementation of effective strategies to address the challenges posed by *bla*NDM-carrying *E. coli*. The findings underscore the urgent need for sustained global collaboration, surveillance efforts, and antimicrobial stewardship to mitigate the impact of these highly resistant strains on public health.

### Electronic supplementary material

Below is the link to the electronic supplementary material.

Supplementary Material 1

## Data Availability

No datasets were generated or analysed during the current study.
